# 

**Published:** 1875-05

**Authors:** 


### TABLE OF CONTENTS.

ORIGINAL COMMUNICATIONS.

Excision of the Upper Extremity of the Femur, after Compound Comminuted Fracture. By P. A. Harris, M.D., Dover, New Jersey... ............ 258
Puerperal Convulsions Treated by the Hypodermic Use of Morphine. By John D. McGill, M.D...... ............... ....... ........ .... 265
Hypodermic Injections. By D. McLean Forman, M.D., Freehold, N. Jersey. 267
A Report of Four Cases of Abscess of the Penis. By E. T. Easly, A.M., M.D., Dallas, Texas............... ..... . ...... ....... ....... 270
A Case of Painless Labor. By Swan M. Burnett, M.D., Knoxville, Tenn.... 272

SELECTIONS.

Varices. By E. H. Toland, M.D., San Francisco................ .......... 274
Cerebro-Spinal Meningitis. By William Reed, M.D., Boston, Mass... .. 276
Practical Observations in Ophthalmology. By J. O. Stilson...... ....... 279
Polypus of the Uterus, Treated by the Internal Administration of Ergot. By Daniel F. Collins, M.D., New York.................................. 286
Progressive Pernicious Anæmia............. ........................ ......... 289

EXTRACTS AND GLEANINGS.

Treatment of Fractures—291. New Method of Treating Typhoid Fever—293. Dr. Paul F. Munde's Treatment of Abortion—294. Tr. Mur. Ferri in Nasal Polypi—295. The Hygiene of the Eyes; Singular case of Malpractice—296. Case of Abstention from Food; Pills of Aloes and Ipecac—297. Action of Mercury upon the Blood; In the Cough of Chronic Phthisis—298. Esmarch's Operation—299. Cincho-Quinine; Chloroform in Congestive Chills—300. New Principles in Surgery—301. Bromide of Potassium in Malarial Fever; A Convenient Local Anodyne—302. Varicose Vein treated by a New Operation; Worms—303. Two Cases of Idiopathic Tetanus; Nitrate of Amyl—304. The Couchard; The "Tractile Method" in Hernia—305. Successful Transplantation of Bone; Remedy for Cholera; Carminative mixture for Young Infants—306. Inunction as a Remedy in Measles and Scarlatina; Transparent Turpentine Liniment; Prolapsus Ani treated by Ergotin—307. Wounds dressed with Silicated Cotton; Explosive Medicines; Dysmenorrhœa—308. Belladonna in the Treatment of Profuse Perspiration; Treatment of Whooping-Cough; Salicylic Acid for Ulcers—309. Milk as a Vehicle of Disease; Chronic Dysentery; Infant Food—310. Bromide of Potassium as a Caustic; A Hint in giving Iodide of Potassium—311. Copaiba in Dropsy; Treatment of Galactorrhœa; Administration of Castor Oil—312. Arrest of Erysipelas by Means of Injections of Carbolic Acid; Ergot in Acute Mania; Trichaniæ in Wild Boars—313. Injection in Gonorrhœa; Treatment of Persistent Neuralgia; Pills of Aconite—314. A Pill for Jaundice; Treatment of Partussis by Inhalation; Liquid Glycerin for Burns—315. Ether for Tape Worm; External Use of Turpentine in the Treatment of Tonsolitis—316.

EDITORIAL AND MISCELLANEOUS.

Charity vs. Policy.................. ...................................... 317
Personal ... ................ ....................... ............. 319
Our Thanks...... ............ ................... ... ............ 319

